# Rehabilitation of post-stroke dysphagia using swallowing exercises: a quasi-experimental pre–post study

**DOI:** 10.3389/fneur.2026.1779315

**Published:** 2026-05-19

**Authors:** Bushra Alshammari, J. Silvia Edison, Awatif Alrasheeday, Sameer A. Alkubati, Sahar Mazied Alshammari, Athar Odah Alshammari, Layla Salem Alshammari, Laila Lafi Alharbi, Hind Mobark Alshamry, Athari Alenazi, Salwa Abd-ElGawad Sallam, Eman Breikan Alshammari, Farhan Alshammari

**Affiliations:** 1 Medical Surgical Nursing Department, College of Nursing, University of Hail, Hail, Saudi Arabia; 2 Medical and Diagnostic Research Center, University of Hail, Hail, Saudi Arabia; 3 Nursing Administration Department, College of Nursing, University of Hail, Hail, Saudi Arabia; 4 Department of Maternity and Child Health, College of Nursing, University of Hail, Hail, Saudi Arabia; 5 Emergency Department, King Salman Specialist Hospital, Hail Health Cluster, Hail, Saudi Arabia; 6 Department of Pharmaceutics, College of Pharmacy, University of Hail, Hail, Saudi Arabia

**Keywords:** Dysphagia Handicap Index, post-stroke dysphagia, quality of life, stroke rehabilitation, swallowing exercises, swallowing rehabilitation

## Abstract

**Background:**

Post-stroke dysphagia is a common and serious complication that negatively affects patient safety, recovery, and quality of life. Swallowing exercises are considered a key rehabilitative approach; however, evidence from real-world clinical settings remains limited.

**Objective:**

This study aimed to evaluate the effectiveness of a swallowing exercise program in improving dysphagia among post-stroke patients.

**Methods:**

A quasi-experimental pre–post design was conducted among 29 post-stroke patients with dysphagia recruited from outpatient follow-up clinics at King Salman Specialist Hospital and King Khaled Hospital, Hail City, Saudi Arabia. Participants received structured swallowing therapy delivered by a specialized physiotherapist, followed by daily home-based exercises. Swallowing difficulty was assessed before and after the intervention using the Dysphagia Handicap Index (DHI).

**Results:**

Post-intervention assessment demonstrated a significant reduction in total DHI scores, indicating improvement in overall swallowing function. Significant improvements were also observed across the functional, physical, and emotional subscales of the DHI.

**Conclusion:**

The findings suggest that swallowing exercises are an effective, feasible, and non-invasive intervention for improving swallowing function and reducing dysphagia-related handicap among post-stroke patients. Incorporating structured swallowing exercises into routine post-stroke rehabilitation may enhance patient outcomes and quality of life.

## 1 Introduction

Stroke is one of the leading causes of morbidity, mortality, and long-term disability worldwide (1). It occurs as a result of an interruption in cerebral blood flow due to vascular occlusion or rupture, leading to ischemic or hemorrhagic brain injury (1). The resulting neuronal damage often produces persistent neurological deficits that significantly impair physical functioning, independence, and overall quality of life among survivors.

One of the most common and clinically significant complications following stroke is dysphagia, a swallowing disorder caused by damage to the central nervous system structures responsible for the coordination of swallowing (2). Post-stroke dysphagia is reported in a substantial proportion of stroke survivors and may occur due to impairment of cortical, subcortical, or brainstem pathways involved in the complex sensorimotor process of swallowing. As swallowing requires the precise integration of multiple cranial nerves and muscle groups, even minor neurological damage can disrupt this process (3).

Dysphagia is associated with serious and potentially life-threatening complications, including aspiration pneumonia, malnutrition, dehydration, and airway obstruction (4). These complications not only increase morbidity and mortality but also prolong hospital stay, increase healthcare costs, and delay functional recovery. In addition, dysphagia has a profound psychosocial impact, as it limits oral intake, reduces the pleasure of eating, and restricts social interaction, thereby negatively affecting patients' emotional wellbeing and quality of life (5).

Early identification and appropriate management of post-stroke dysphagia are therefore essential components of comprehensive stroke rehabilitation (6). Conventional management strategies include dietary texture modification, compensatory swallowing techniques, and postural adjustments aimed at improving swallowing safety. However, such approaches primarily focus on compensation rather than functional recovery of the swallowing mechanism.

Swallowing exercises represent a rehabilitative approach that targets the underlying physiological deficits by strengthening the muscles involved in swallowing, improving neuromuscular coordination, and promoting neural reorganization (7). These exercises are non-invasive, cost-effective, and can be easily administered in clinical and rehabilitation settings. Emerging evidence suggests that structured swallowing exercise programs may lead to meaningful improvements in swallowing function and reduce the severity of dysphagia in post-stroke patients. While recent RCTs and meta-analyses (8, 9) support the efficacy of swallowing exercises, evidence from real-world clinical settings—particularly in Middle Eastern populations without prior dysphagia intervention—remains limited. This study addresses this gap by evaluating a pragmatic, combined supervised and home-based exercise program, with attention to multidimensional patient-reported outcomes including emotional wellbeing.

Given the high prevalence and serious consequences of post-stroke dysphagia, there is a need for further interventional studies to evaluate the effectiveness of swallowing exercises in improving swallowing outcomes. Therefore, the present study aims to assess swallowing function before and after a structured swallowing exercise intervention among post-stroke patients, in order to determine whether this rehabilitative strategy leads to significant improvement in dysphagia severity.

### 1.1 Objectives of the study

- To assess the level of dysphagia among post-stroke patients prior to the implementation of swallowing exercises.
- To assess the level of dysphagia among post-stroke patients after the implementation of swallowing exercises.
- To compare pre-intervention and post-intervention swallowing function in post-stroke patients following a structured swallowing exercise program.

### 1.2 Significance of the study

Post-stroke dysphagia is a common and serious complication that significantly affects patient safety, recovery, and quality of life (10). If not appropriately managed, it may lead to aspiration pneumonia, malnutrition, dehydration, prolonged hospitalization, and increased healthcare costs (11). Despite the availability of various management strategies, there remains a need for effective, low-cost, and non-invasive interventions that can be easily implemented in routine clinical practice (12, 13).

This study is significant as it provides empirical evidence on the effectiveness of swallowing exercises as a rehabilitative intervention for post-stroke dysphagia (12). By employing a pre–post interventional design, the study contributes to understanding whether structured swallowing exercises can produce measurable improvements in swallowing function within a short intervention period.

The findings of this study may support evidence-based clinical decision-making by nurses and rehabilitation professionals and encourage the integration of swallowing exercises into standard post-stroke care. Furthermore, the results may help reduce the incidence of dysphagia-related complications, enhance patient safety, and improve overall quality of life for stroke survivors. At a broader level, the study contributes to the development of cost-effective rehabilitation strategies and supports the optimization of stroke care services.

### 1.3 Hypotheses

H1: There is a statistically significant reduction in the level of dysphagia among post-stroke patients after the implementation of swallowing exercises compared to before the intervention at the 0.05 level of significance.

## 2 Materials and methods

### 2.1 Design

A quasi-experimental non-equivalent pre–post test design was employed to evaluate the effect of swallowing exercises on dysphagia among post-stroke patients. Swallowing function was assessed before and after the intervention, with participants serving as their own controls. Due to practical and ethical considerations, randomization was not applied. Participants were recruited using convenience sampling from the selected hospital setting.

### 2.2 Setting

The study was conducted at King Salman Specialist Hospital and King Khaled Hospital in Hail City, Kingdom of Saudi Arabia. These hospitals are major tertiary healthcare institutions that provide comprehensive medical and rehabilitative services to patients with neurological disorders, including stroke. Both facilities serve a large and diverse population of stroke survivors, making them suitable settings for conducting clinical rehabilitation research.

Participants were recruited from the outpatient follow-up clinics, where post-stroke patients attend regular visits for ongoing medical management, rehabilitation monitoring, and secondary prevention. Recruiting patients from follow-up clinics allowed the identification of clinically stable individuals who were able to participate in the swallowing exercise intervention and complete pre- and post-intervention assessments.

### 2.3 Sample

The study sample consisted of 29 post-stroke patients diagnosed with dysphagia who attended the outpatient follow-up clinics at King Salman Specialist Hospital and King Khaled Hospital in Hail City, Kingdom of Saudi Arabia. Participants were recruited using a convenience sampling technique based on their availability and eligibility during the data collection period.

Eligible participants were adults in the post-stroke phase who were clinically stable, cognitively capable of understanding and following instructions, and willing to participate in the study, as determined by the treating physician. Patients with acute medical instability, those receiving exclusive enteral or parenteral nutrition, and individuals with significant cognitive or communication impairments that could interfere with their ability to engage effectively in the intervention were excluded.

### 2.4 Recruitment procedure

Participant recruitment was conducted through the outpatient follow-up clinics at King Salman Specialist Hospital and King Khaled Hospital. The researchers initially approached the clinic gatekeepers (healthcare staff responsible for patient coordination) to facilitate access to potentially eligible post-stroke patients.

Potential participants who met the eligibility criteria were provided with detailed information about the study, including its purpose, procedures, duration, and their rights as participants. An information sheet, written informed consent form, and consent-to-be-contacted form were given to each eligible patient. Adequate time was allowed for patients to read the materials and ask questions before making a decision regarding participation.

Patients who agreed to participate and provided written consent were subsequently contacted by the research team to arrange the study procedures. During this stage, the researchers clarified any additional details related to the intervention and assessments and addressed participants' questions or concerns prior to data collection.

### 2.5 Intervention and procedure

The intervention consisted of a structured swallowing exercise program designed to improve the strength, coordination, and control of the muscles involved in swallowing among post-stroke patients with dysphagia. Swallowing function was assessed before and after the intervention using the Dysphagia Handicap Index (DHI) to evaluate changes in swallowing difficulty over time.

At baseline (pre-intervention), participants' swallowing difficulty was measured using the DHI questionnaire. Following baseline assessment, participants were enrolled in the swallowing exercise intervention, which was implemented over a 3-month period. Post-intervention swallowing function was reassessed at the end of the intervention period using the same instrument to determine changes in dysphagia severity.

#### 2.5.1 Swallowing exercise program

The swallowing exercise program was designed as a rehabilitative, physiology-based intervention targeting impairments across the oral and pharyngeal phases of swallowing. The program incorporated oromotor exercises focusing on lip closure, lip retraction and protrusion, cheek puffing, and jaw mobility to enhance oral seal, bolus control, and coordination during the oral preparatory and propulsive phases (14). In addition, tongue-based swallowing exercises, including the Masako maneuver, were used to strengthen the posterior tongue and pharyngeal constrictor muscles, thereby improving tongue base retraction and posterior pharyngeal wall movement during swallowing. The program also included the Mendelsohn maneuver, which aims to prolong hyolaryngeal elevation and enhance upper esophageal sphincter opening, contributing to improved airway protection and bolus transit through the pharynx (15).

#### 2.5.2 Protocol and dosing

To enhance the reproducibility and clinical applicability of the intervention, the swallowing exercise program was operationalized using a structured dosing framework. Each 30-min session consisted of a standardized sequence of exercises targeting oromotor control and pharyngeal strengthening. Oromotor exercises (lip closure, lip protrusion/retraction, cheek puffing, and jaw mobility) were performed in 2–3 sets of 8–12 repetitions per exercise, focusing on controlled, deliberate movements to improve oral preparatory function. Tongue-based exercises, including the Masako maneuver, were performed in 2 sets of 5–10 repetitions, with each repetition involving sustained posterior tongue engagement to facilitate pharyngeal wall contact. The Mendelsohn maneuver was practiced in 2 sets of 5 repetitions, with each repetition involving voluntary prolongation of hyolaryngeal elevation for approximately 3–5 s to enhance upper esophageal sphincter opening. Rest intervals of approximately 30–60 s were allowed between sets to prevent fatigue and ensure safe performance. The overall session structure was maintained consistently across supervised and home-based sessions to standardize exposure to the therapeutic stimulus.

#### 2.5.3 Frequency, progression, adherence

Participants engaged in the swallowing exercise program on a daily basis throughout the 3-month intervention period, combining supervised physiotherapist-led sessions with independent home practice. To account for inter-individual variability in tolerance and recovery stage, a principle of gradual progression was applied, whereby exercise repetitions and hold durations were adjusted incrementally based on patient performance, fatigue level, and safety considerations, as assessed during follow-up interactions. While the total daily exercise duration was maintained at approximately 30 min, participants demonstrating improved control were encouraged to increase repetition consistency and precision rather than overall session length. Adherence to the prescribed protocol was supported through the use of a structured home exercise log, where participants recorded session completion, duration, and any experienced difficulty. These logs were periodically reviewed by the research team to monitor compliance and reinforce correct technique. This structured yet flexible dosing approach was designed to balance standardization with patient-centered adaptation, thereby optimizing both feasibility and therapeutic effectiveness.

#### 2.5.4 Intervention delivery and follow-up

Participants received swallowing therapy sessions delivered by a specialized physiotherapist, with each session lasting 30 min. During these sessions, the physiotherapist demonstrated the prescribed swallowing exercises, followed by supervised patient practice to ensure correct technique and safe performance. Participants' execution of the exercises, understanding of instructions, and adherence were carefully observed and recorded. Once participants demonstrated adequate competence in performing the swallowing exercises correctly, they were instructed to continue the exercise program independently at home. Participants were advised to perform the swallowing exercises daily for 30 min throughout the intervention period.

To support adherence and self-monitoring, participants were provided with a home exercise log, in which they recorded the duration of each exercise session, whether the exercises were performed each day, and any difficulties or discomfort experienced during practice. These records were reviewed during follow-up to monitor compliance and identify potential barriers to exercise performance.

#### 2.5.5 Outcome measure: Dysphagia Handicap Index (DHI)

Swallowing difficulty was assessed using the DHI, a validated self-reported questionnaire designed to evaluate the perceived impact of dysphagia on an individual's daily functioning and quality of life (16). The DHI is widely utilized in both clinical practice and research settings to assess the severity of dysphagia, particularly among patients with neurological conditions such as stroke. It provides a comprehensive measure of how swallowing difficulties affect functional abilities, physical symptoms, and emotional wellbeing.

The DHI consists of 25 items distributed across three distinct subscales. The functional subscale comprises nine items and focuses on the impact of dysphagia on eating habits, the need for dietary modifications, and participation in social activities. This domain reflects the extent to which swallowing difficulties interfere with routine daily activities and social interactions involving food. The physical subscale also includes nine items and evaluates the physical manifestations of swallowing difficulties. These items address symptoms such as coughing or choking during meals, sensations of food sticking in the throat, and unintended weight loss, thereby capturing the physiological burden associated with dysphagia. The emotional subscale consists of seven items and measures the psychological and emotional consequences of dysphagia. This domain explores feelings of anxiety, embarrassment, frustration, and fear related to eating, highlighting the emotional distress that may accompany persistent swallowing problems.

Each item in the DHI is rated using a three-point Likert scale, with responses scored as 0 for no problem, 2 for a moderate problem, and 4 for a severe problem. The total DHI score ranges from 0 to 100, with higher scores indicating a greater perceived severity of swallowing difficulty and associated handicap. Subscale scores are calculated by summing the item scores within each domain, allowing for a detailed analysis of the functional, physical, and emotional dimensions of dysphagia. For interpretative purposes, DHI total scores were categorized as mild (0–30), moderate (31–59), or severe (60–100), following the severity pattern observed in the original validation study by Silbergleit et al. (16) and subsequent adaptations. In this study, the DHI was administered before the initiation of the swallowing exercise intervention and after completion of the intervention period to evaluate changes in swallowing difficulty. Differences between pre- and post-intervention DHI scores were used to determine the effectiveness of the swallowing exercise program. The DHI has demonstrated good validity, reliability, and responsiveness to therapeutic interventions, making it an appropriate outcome measure for assessing changes in swallowing function in post-stroke patients (17). The study additionally collected sociodemographic and clinical data from all participants using a structured questionnaire. Sociodemographic variables included age, gender, educational level, employment status, and prior receipt of information related to swallowing therapy. Clinical variables included previous history of stroke, time since stroke onset, and duration of dysphagia.

### 2.6 Ethical considerations

Ethical approval for the study was obtained from the Institutional Review Board (IRB) of the University of Hail (Approval No. N-239-5439), granted in June 2024. Official permission was also secured from the administrative authorities of King Salman Specialist Hospital and King Khaled Hospital prior to participant recruitment and data collection. All participants were fully informed about the purpose of the study, the nature of the intervention, and the procedures involved. Written informed consent was obtained from each participant before enrollment. Participation was entirely voluntary, and participants were informed of their right to withdraw from the study at any time without any effect on their medical care.

Confidentiality and anonymity were strictly maintained throughout the study. Participants were assigned unique identification codes, and no personal identifiers were included in the data analysis or reporting. All collected data were securely stored and accessed only by the research team. The intervention involved non-invasive swallowing exercises and posed minimal risk to participants. Safety precautions were observed during all intervention sessions, and participants were closely monitored for any discomfort or adverse events. The study was conducted in accordance with ethical principles outlined in the Declaration of Helsinki.

### 2.7 Data analysis

SPSS version 27 was used to analyse data. The patient characteristics and clinical data of participants were summarized using descriptive statistics such as means, standard deviations, frequencies, and percentages. Prior to inferential analysis, the normality of continuous variables (pre–post differences in DHI scores) was assessed using the Shapiro–Wilk test (*p* > 0.05) and visual inspection of Q–Q plots. The assumptions for parametric testing were considered adequately met. Paired *t*-tests were conducted to compare pre-intervention and post-intervention DHI total and subscale scores. To account for multiple comparisons across the three DHI subscales (functional, physical, and emotional), a Bonferroni correction was applied, resulting in an adjusted significance level of *p* < 0.017. The total DHI score was considered the primary outcome and was therefore evaluated using the conventional significance level of *p* < 0.05. To enhance the robustness of interpretation, effect sizes (Cohen's d) and 95% confidence intervals were reported alongside *p-values*. A *p-value* of < 0.05 indicated statistical significance.

## 3 Results

A total of 29 participants were included in the study (Table 1). Most participants were aged 51–60 years (44.8%), followed by those aged 40–50 years and 61–70 years (17.2% each). The sample was nearly equally distributed by gender, with females representing 51.7% and males 48.3%. Regarding educational level, the majority had completed primary school education (62.1%), while 37.9% had a high school education. Most participants were retired (65.5%), whereas 34.5% were unemployed. Notably, none of the participants reported having previously received information related to dysphagia (100%).

**Table 1:** Sociodemographic characteristics of participants (*N* = 29).

Variables	Categories	*n*	%
Age	40–50	5	17.2
	51–60	13	44.8
	61–70	5	17.2
Gender	Male	14	48.3
	Female	15	51.7
Educational level	Primary school	18	62.1
	High school	11	37.9
Employment status	Unemployed	10	34.5
	Retired	19	65.5

Table 2 illustrates the clinical data of participants; most participants experienced a first-ever stroke (86.2%), while a smaller proportion had a history of previous stroke (13.8%). The onset of stroke was less than 1 year prior to the majority of participants (86.2%). Dysphagia was reported to have started at the time of stroke onset in most cases (93.1%), whereas only 6.9% reported a duration of dysphagia of less than 6 weeks. Notably, a high proportion of patients were classified as having severe dysphagia (62.1%), followed by moderate dysphagia (37.9%), while no patients fell into the mild category.

**Table 2:** Clinical data of participants (*N* = 29).

Variables	Categories	*n*	%
Previous history of stroke	First-ever stroke	25	86.2
	Previous stroke	4	13.8
Onset of stroke	Less than 1 year ago	25	86.2
	More than or equal to 1 year ago	4	13.8
Duration of dysphagia	Since the stroke began	27	93.1
	Less than 6 weeks	2	6.9
Severity of dysphagia	Mild (0–30)	0	00
	Moderate (31–59)	11	37.9
	Severe (60–100)	18	62.1

As shown in Table 3, paired *t*-test analysis revealed significant improvements in patients' DHI scores following the intervention. The total DHI score showed a significant reduction (mean difference = 4.66, 95% CI: 2.90 to 6.42, *p* < 0.001), with a large effect size (Cohen's *d* = 1.376), indicating a substantial overall improvement in perceived dysphagia severity. At the subscale level, the emotional domain exhibited the greatest improvement (mean difference = 2.21, 95% CI: 1.50 to 2.92, *p* < 0.001), with a very large effect size (*d* = 1.981), reflecting a pronounced reduction in dysphagia-related emotional burden. The physical domain also showed a significant reduction (mean difference = 1.90, 95% CI: 0.90 to 2.90, *p* < 0.001), with a large effect size (*d* = 0.834), suggesting meaningful improvement in swallowing-related physical symptoms. After applying the Bonferroni-adjusted significance threshold (*p* < 0.017) for subscale comparisons, the reductions in the physical (*p* < 0.001) and emotional (*p* < 0.001) domains remained statistically significant. However, the improvement observed in the functional domain (*p* = 0.018) did not retain statistical significance after adjustment. Despite this, the functional domain demonstrated a moderate effect size (Cohen's *d* = 0.466), suggesting a potentially meaningful but less robust change.

**Table 3:** Patients' Dysphagia Handicap Index (DHI) scores pre- and post-intervention (*N* = 29).

DHI domain	Pre-intervention Mean ±SD	Post-intervention Mean ±SD	Effect size (d)	95% CI	p-value^*^
Functional	27.03 ± 9.00	26.48 ± 8.85	0.466	0.079–0.846	0.018
Physical	26.55 ± 8.71	24.65 ± 9.99	0.834	0.405–1.253	< 0.001
Emotional	19.65 ± 6.88	17.44 ± 6.75	1.981	1.343–2.606	< 0.001
**Total**	73.24 ± 21.70	68.58 ± 22.32	1.376	0.859–1.880	< 0.001

^*^Paired t-test, SD, Standard deviation; CI, Confidence interval.

Bonferroni-adjusted significance level for subscales set at p < 0.017; total score treated as the primary outcome.

## 4 Discussion

This quasi-experimental study suggested significant improvements in self-reported dysphagia handicap after 3 months of combined supervised and home-based swallowing exercises. The DHI total score decreased (mean ~5-point reduction), indicating a lower perceived swallowing-related disability post-intervention. Importantly, all three DHI subscales—physical, functional, and emotional—showed significant gains. The physical domain improvement suggests patients experienced fewer swallowing symptoms and less physical difficulty with eating and drinking. The functional domain also improved, reflecting better performance in daily activities involving feeding, perhaps through greater dietary autonomy or reduced need for compensations. Notably, the emotional domain exhibited the greatest improvement, highlighting a substantial reduction in psychological distress related to dysphagia. This likely means patients felt less anxious or fearful about choking and less embarrassed or socially isolated during meals. The broad pattern of DHI improvement across domains underscores a meaningful enhancement in swallowing-related quality of life. Given that the DHI is a validated 25-item index covering functional, physical, and emotional aspects of dysphagia impact (18), the post-therapy score changes in our study indicate that swallowing rehabilitation had a positive, holistic effect on patients' wellbeing. These findings suggest that even a 3-month regimen of targeted exercises can yield clinically relevant benefits, reducing the handicap of post-stroke dysphagia in multiple dimensions.

Our results align with a growing body of evidence supporting the effectiveness of swallowing exercises in post-stroke dysphagia. Recent clinical trials have similarly reported significant improvements in patient-reported outcomes following dysphagia therapy. For example, Alyanak et al. (18), conducted a randomized trial in stroke survivors and found that both a conventional exercise group and an augmented biofeedback group showed significant pre–post improvement in DHI total scores and subscales, with greater gains in the biofeedback-enhanced therapy group. Additionally, objective swallowing function measures in the literature often improve alongside DHI/QoL scores. In the trial by Alyanak et al. (18), the exercise group showed better Functional Oral Intake Scale (FOIS) scores and swallowing safety (Penetration-Aspiration Scale) compared to controls, and a portion of feeding-tube-dependent patients regained oral feeding ability after therapy. Similarly, a systematic review of stroke rehabilitation noted that behavioral swallowing interventions can improve swallowing function and facilitate resumption of oral intake (8). Our study did not directly measure dietary status or aspiration, but the significant DHI-physical improvements imply parallel gains in swallowing safety and efficiency, which is corroborated by such objective outcomes in prior studies.

Beyond individual trials, recent high-level evidence supports dysphagia rehabilitation as a key contributor to better outcomes in stroke. A 2023 systematic review on quality of life in post-stroke dysphagia concluded that dysphagia therapy significantly improves stroke survivors' QoL (19). The authors found that patients' overall wellbeing was better after swallowing treatment, echoing earlier observations that treating dysphagia can raise QoL scores (including DHI) by alleviating the burden of eating difficulties.

Our findings reinforce this: the sizable DHI score reductions we observed are indicative of improved health-related QoL, consistent with the broader literature. They also mirror foundational studies which reported that intensive swallowing therapy can restore more normal eating and reduce dysphagia severity. For instance, rehabilitative exercise programs (both direct maneuvers like effortful swallow, and indirect exercises like the Shaker head-lift) have been shown to reduce dysphagia severity and symptoms in stroke patients. Such exercises strengthen the swallowing musculature and improve bolus clearance, translating into better functional swallowing and patient perceptions. In fact, previous systematic reviews and meta-analyses have demonstrated that specific exercise regimens (e.g., chin tuck against resistance or Shaker exercises) significantly improve biomechanical aspects of swallowing such as hyolaryngeal excursion and airway protection, ultimately enhancing swallowing safety (9, 20). Taken together, the improvements observed in our study are well substantiated by contemporary research: swallowing exercises result in better clinical swallowing outcomes and a reduction in the handicapping effects of dysphagia.

It is also noteworthy that the magnitude of improvement in our quasi-experimental study is comparable to or greater than what might be expected from spontaneous recovery alone, especially given that many participants were in the chronic phase of stroke. Dysphagia recovery without intervention tends to plateau after the acute/subacute period (often within the first few months post-stroke), after which persistent dysphagia is less likely to resolve on its own. Our intervention spanned 3 months, by which time natural recovery would have slowed, yet we still observed significant gains—reinforcing that the exercises provided therapeutic benefit beyond any spontaneous healing. This observation aligns with intensive rehabilitation being a key factor: a recent systematic review of RCTs noted that the intensity of swallowing therapy has a positive effect on dysphagia recovery (12). In other words, patients who engage in frequent, targeted swallowing practice (as in our supervised plus home exercise program) tend to achieve better swallowing outcomes. Consistent with this, current clinical guidelines advocate for active dysphagia rehabilitation in stroke care. The European Stroke Organization and European Society for Swallowing Disorders joint guideline (21) recommends that stroke patients with dysphagia receive swallowing therapy and that interventions be started early and intensively when possible, although it acknowledges the evidence base is still developing. Our findings add support to these recommendations by demonstrating tangible QoL benefits of such therapy. In sum, the positive outcomes in DHI scores observed here are in agreement with both recent clinical studies and expert guidelines, all of which underscore swallowing rehabilitation as an effective means to mitigate the deficits and dangers of post-stroke dysphagia.

Notably, a small subset of participants in this study (*n* = 4) were in the chronic phase of stroke (≥1 year post-onset), a stage at which spontaneous recovery of swallowing function is typically limited. Despite this, these participants demonstrated improvements in DHI scores consistent in direction with those observed in the overall cohort. This finding suggests that structured swallowing exercises may retain therapeutic value beyond the conventional window of neurorecovery. The observed improvements could be attributed to mechanisms such as targeted neuromuscular strengthening, improved coordination of swallowing musculature, and potential activity-dependent neuroplasticity, even in the chronic stage. However, given the small number of chronic participants, these findings should be interpreted with caution and cannot be considered conclusive. Future research with adequately powered samples and subgroup-specific analyses is warranted to further explore the efficacy and optimal design of swallowing rehabilitation protocols in chronic post-stroke populations.

### 4.1 Addressing the emotional, functional, and physical impact of dysphagia

The improvements across DHI subscales in our study carry important implications for the multidimensional impact of dysphagia. Physically, post-stroke dysphagia can lead to serious health complications—including malnutrition, dehydration, and aspiration pneumonia—which increase morbidity and mortality (18). By improving the physical domain of DHI, our exercise program likely helped patients swallow more safely and effectively, thereby potentially reducing these risks. Patients may have experienced fewer incidents of coughing or choking and improved nutritional intake, which is critical because dysphagia's physical burden (weight loss, pulmonary infections, etc.) also directly affects survival and overall recovery. Even though we did not measure pneumonia rates or nutritional status in this study, the significant physical DHI gains suggest a trend toward safer swallowing. This is consistent with other reports where therapy led to objective improvements —for example, better airway protection on instrumental swallow exams or transitions from tube feeding to oral diets. As swallowing function improves, patients can handle oral intake more safely, which in turn improves their strength and health, creating a positive feedback loop on the physical aspect of recovery.

Functionally, dysphagia often impairs the ability to maintain independence and social participation. Stroke survivors with dysphagia may require modified diets, feeding assistance, or prolonged mealtimes, all of which impede their daily routines and reduce autonomy. In our study, the functional subscale of the DHI (which covers the impact on daily activities, work, and social life) showed a statistically significant improvement. Although the effect size was modest relative to other domains, even a moderate functional gain is noteworthy—it might mean, for instance, that a patient progressed from needing pureed foods to tolerating soft solids, or became able to dine out with family whereas before they felt unable. Such changes greatly enhance a person's day-to-day experience. Prior research illustrates that dysphagia severity correlates with worse social functioning and role limitations (19); therefore, alleviating dysphagia should improve those aspects of quality of life. Indeed, one longitudinal study observed that as rehabilitation therapy progressed, patients' DHI scores improved in tandem with better feeding mode (oral vs. non-oral feeding) (22). Our results resonate with this finding: by the end of our program, patients perceived less functional handicap, likely because they could more freely choose foods and engage in meals with greater normalcy. It is important to acknowledge that the functional domain improved slightly less than the others; this could be because some chronic stroke patients still had dietary restrictions or needed compensatory strategies post-therapy. Full restoration of unrestricted diet or complete independence might require longer treatment or adjunctive strategies. Nonetheless, even incremental functional improvements can reduce caregiver burden and increase patients' self-sufficiency, underlining the real-world value of swallowing exercises.

Emotionally, dysphagia's impact is profound and often under-recognized. Many patients experience anxiety (e.g., fear of choking or of eating in public), embarrassment, and even depression due to their swallowing difficulties. They may avoid eating with others or feel a loss of pleasure in food, leading to social withdrawal. In our study, the emotional domain of the DHI improved the most dramatically. This suggests the intervention not only improved the act of swallowing but also patients' confidence and psychological outlook. With safer swallowing skills, patients likely felt less afraid of aspirating or suffocating during meals. They may have also regained a sense of normalcy and dignity at mealtimes, reducing feelings of embarrassment or social isolation. The literature strongly supports this link between dysphagia improvement and emotional wellbeing. Dysphagia is known to detrimentally affect psychosocial wellbeing, causing increased emotional distress and social isolation in stroke survivors (19). Conversely, when dysphagia is effectively treated, patients report reductions in stress and improved mental health (24). Our findings exemplify this: after therapy, patients' emotional handicap scores dropped significantly, reflecting reduced psychological burden. This outcome is especially important because emotional health can influence overall stroke recovery—a less anxious, more socially engaged patient is likely to participate better in rehabilitation and have a higher quality of life. Thus, the large gains in the DHI-emotional subscale highlight a key benefit of swallowing rehabilitation: it can restore not just physical function but also alleviate the fear and frustration that accompany post-stroke dysphagia. Clinicians should consider this psychological dimension; even simple encouragement from seeing progress in swallowing ability can boost a patient's mood and motivation. In summary, our intervention addressed all three impact areas of dysphagia. The reduction in physical risks, enhancement of functional eating ability, and alleviation of emotional strain together paint a picture of a therapy that meaningfully improves patients' lives. These multi-faceted benefits reinforce the value of treating dysphagia comprehensively, as recommended by current rehabilitation frameworks that emphasize both the safety and the quality-of-life aspects of swallowing treatment (8). However, the magnitude of effect sizes may be overestimated due to the small sample and potential self-report bias, including response shift and expectancy effects.

### 4.2 Limitations

While our study suggests promising outcomes, there are limitations that must temper the interpretation of the results. First, as a quasi-experimental (pre–post) design without a randomized control group, we cannot definitively attribute all improvements to the intervention. It is encouraging that the magnitude of DHI improvement exceeds what is typically expected from spontaneous recovery in the chronic post-stroke phase (23), but a controlled trial would strengthen causal inference. Future studies should include a comparison group (e.g., a no-treatment or conventional care control) to account for potential placebo effects or natural recovery. Second, although all participants were clinically stable and recruited from outpatient follow-up clinics—indicating that they had progressed beyond the acute phase of stroke and were considered suitable for participation in rehabilitation interventions—stroke type and lesion location were not systematically documented based on patients' medical records. As these factors may significantly influence functional recovery and response to swallowing therapy, their absence limits the ability to stratify patients and fully interpret variability in treatment outcomes.

Third, our sample size (*N* = 29) was relatively modest. Although the effects reached statistical significance, a larger sample in future research would increase the power to detect more subtle improvements (especially in the functional domain) and improve generalizability. Third, our outcome focus was on patient-reported handicap DHI with no direct instrumental assessments of swallowing physiology or functional diet changes recorded. It would be valuable to include objective measures such as Videofluoroscopic Swallowing Study-based scores, penetration-aspiration ratings, or nutritional intake levels in subsequent studies. These would corroborate the self-reported improvements with concrete functional data and help determine if the observed QoL gains correspond to measurable reductions in aspiration or diet restrictions. For instance, documenting how many patients upgraded their diet consistency or discontinued tube feeding would provide clinical context to the DHI changes.

In addition, the lack of detailed clinical characterization of dysphagia should be considered when interpreting the study findings. Participants were not stratified according to the specific type of dysphagia (e.g., oral, pharyngeal, or mixed), which may differentially influence responsiveness to targeted swallowing exercises. Furthermore, information regarding the anatomical location of the stroke lesion was not available, despite its recognized role in determining the severity and pattern of swallowing impairment. These factors may act as potential confounders and could partially explain variability in treatment response among participants. Future studies are encouraged to incorporate detailed clinical and neuroanatomical profiling to allow for more precise interpretation of intervention outcomes and to support the development of tailored rehabilitation protocols.

Additionally, the intervention lasted 3 months with assessments immediately post-therapy; we do not know if these improvements persist long-term. Longer-term follow-up is recommended to see if patients maintain their gains or require booster sessions to prevent regression. The durability of dysphagia therapy effects is an area needing more evidence—some studies suggest that without ongoing practice, some strength gains can diminish, whereas others show sustained benefits at 6 or 12 months (12). Investigating maintenance strategies (e.g., continued home exercises beyond supervised therapy) could be fruitful. We also acknowledge that our exercise program was fairly labor-intensive (regular supervised sessions plus daily home practice), which may not be accessible to all patients. Adherence to home exercises can wane over time. In our study, adherence was encouraged through periodic supervision, but we did not formally measure compliance. Future work might incorporate technology (for example, mobile apps or biofeedback devices) to monitor and promote adherence, which could enhance outcomes as suggested by emerging research. Finally, the use of self-reported outcomes and lack of objective swallowing assessments may introduce bias and limit the accuracy of effect size estimates.

## 5 Conclusions

This quasi-experimental study provides clear evidence that a structured swallowing exercise program can significantly reduce dysphagia among post-stroke patients. Following a 3-month intervention combining supervised physiotherapist-led sessions with daily home-based exercises, participants demonstrated statistically significant improvements in overall swallowing function, as reflected by reductions in total DHI scores. Importantly, meaningful gains were observed across the physical, functional, and emotional domains, indicating that the benefits of the intervention extended beyond physiological swallowing mechanics to encompass daily functioning and psychosocial wellbeing.

The marked improvement in the physical domain suggests enhanced swallowing safety and efficiency, which may translate into reduced risk of complications such as aspiration, malnutrition, and dehydration. Functional improvements indicate greater independence and participation in eating-related activities, while the substantial reduction in emotional distress highlights the intervention's positive impact on patients' confidence, anxiety, and social engagement during meals. Collectively, these multidimensional improvements underscore the value of swallowing rehabilitation as a holistic intervention that addresses both clinical outcomes and quality of life in stroke survivors.

From a clinical perspective, the findings support the integration of structured swallowing exercises into routine post-stroke rehabilitation programs. The intervention is non-invasive, relatively low-cost, and feasible to implement in outpatient and community settings, particularly when combined with home-based practice. Given that none of the participants had previously received formal dysphagia-related education or therapy, the observed improvements also emphasize the importance of early referral and patient education as part of comprehensive stroke care. Despite its strengths, the study's quasi-experimental design, modest sample size, and reliance on self-reported outcomes warrant cautious interpretation. Future research employing randomized controlled designs, larger and more diverse samples, objective instrumental swallowing assessments, and longer follow-up periods is recommended to confirm causality, assess long-term sustainability of gains, and further elucidate the mechanisms underlying improvement.

In conclusion, structured swallowing exercises represent an effective and practical rehabilitative strategy for reducing the burden of post-stroke dysphagia. Their incorporation into standard rehabilitation pathways has the potential to enhance patient safety, promote functional recovery, and significantly improve the quality of life of stroke survivors.

## Data Availability

The raw data supporting the conclusions of this article will be made available by the authors, without undue reservation.
