# Correction: Rehabilitation of post-stroke dysphagia using swallowing exercises: a quasi-experimental pre–post study

**DOI:** 10.3389/fneur.2026.1907983

**Published:** 2026-07-01

**Authors:** Bushra Alshammari, J. Silvia Edison, Awatif Alrasheeday, Sameer A. Alkubati, Sahar Mazied Alshammari, Athar Odah Alshammari, Layla Salem Alshammari, Laila Lafi Alharbi, Hind Mobark Alshamry, Athari Alenazi, Salwa Abd-ElGawad Sallam, Eman Breikan Alshammari, Farhan Alshammari

**Affiliations:** 1 Medical Surgical Nursing Department, College of Nursing, University of Hail, Hail, Saudi Arabia; 2 Medical and Diagnostic Research Center, University of Hail, Hail, Saudi Arabia; 3 Nursing Administration Department, College of Nursing, University of Hail, Hail, Saudi Arabia; 4 Department of Maternity and Child Health, College of Nursing, University of Hail, Hail, Saudi Arabia; 5 Emergency Department, King Salman Specialist Hospital, Hail Health Cluster, Hail, Saudi Arabia; 6 Department of Pharmaceutics, College of Pharmacy, University of Hail, Hail, Saudi Arabia

**Keywords:** Dysphagia Handicap Index, post-stroke dysphagia, quality of life, stroke rehabilitation, swallowing exercises, swallowing rehabilitation

Affiliation Medical and Diagnostic Research Center, University of Hail, Hail, Saudi Arabia was omitted from the paper. This affiliation has now been added for author(s) Bushra Alshammari, Salwa Abd-ElGawad Sallam, and Farhan Alshammari.

The original version of this article has been updated.

